# BST2/Tetherin Inhibition of Alphavirus Exit

**DOI:** 10.3390/v7042147

**Published:** 2015-04-22

**Authors:** Yaw Shin Ooi, Mathieu Dubé, Margaret Kielian

**Affiliations:** Department of Cell Biology, Albert Einstein College of Medicine, 1300 Morris Park Ave., Bronx, NY 10461, USA; E-Mails: ysooi@stanford.edu (Y.S.O.); Mathieu.Dube@iaf.inrs.ca (M.D.)

**Keywords:** alphavirus, tetherin/BST2, virus budding, rubella virus, dengue virus

## Abstract

Alphaviruses such as chikungunya virus (CHIKV) and Semliki Forest virus (SFV) are small enveloped RNA viruses that bud from the plasma membrane. Tetherin/BST2 is an interferon-induced host membrane protein that inhibits the release of many enveloped viruses via direct tethering of budded particles to the cell surface. Alphaviruses have highly organized structures and exclude host membrane proteins from the site of budding, suggesting that their release might be insensitive to tetherin inhibition. Here, we demonstrated that exogenously-expressed tetherin efficiently inhibited the release of SFV and CHIKV particles from host cells without affecting virus entry and infection. Alphavirus release was also inhibited by the endogenous levels of tetherin in HeLa cells. While rubella virus (RuV) and dengue virus (DENV) have structural similarities to alphaviruses, tetherin inhibited the release of RuV but not DENV. We found that two recently identified tetherin isoforms differing in length at the N-terminus exhibited distinct capabilities in restricting alphavirus release. SFV exit was efficiently inhibited by the long isoform but not the short isoform of tetherin, while both isoforms inhibited vesicular stomatitis virus exit. Thus, in spite of the organized structure of the virus particle, tetherin specifically blocks alphavirus release and shows an interesting isoform requirement.

## 1. Introduction

Alphaviruses are small enveloped plus-sense RNA viruses that include important human pathogens such as chikungunya virus (CHIKV) and Venezuelan equine encephalitis virus (VEEV) [1]. The alphavirus genus is in the Togaviridae family, which also includes the rubivirus rubella virus (RuV). Alphaviruses such as CHIKV and the encephalitic alphaviruses are listed as priority pathogens by the Centers for Disease Control because of their emerging nature, serious effects on human health, and potential to be exploited as biological weapons. Most alphaviruses are arthropod-borne and thus cycle in nature between vertebrate hosts and arthropod vectors such as mosquitoes. There are no licensed antivirals for the alphaviruses, and an approved vaccine is currently available only for VEEV [2,3].

Alphaviruses are structurally well-defined viruses with a roughly spherical shape [1,4,5]. The nucleocapsid contains one copy of the genomic RNA enclosed in a shell formed by 240 copies of the capsid protein. This core is enveloped by a lipid bilayer studded with 240 copies of the viral envelope glycoproteins E2, which mediates receptor binding, and E1, the membrane fusion protein [6,7]. Both the nucleocapsid and the outer envelope protein layer are organized with T = 4 icosahedral quasi-symmetry. The envelope protein lattice almost completely covers the virus membrane, with limited exposure of the lipid bilayer.

Alphaviruses infect host cells by receptor-mediated endocytosis and a low pH-triggered membrane fusion reaction that releases the nucleocapsid into the cytoplasm [8,9,10]. RNA replication and viral protein synthesis take place in the cytoplasm, and the envelope proteins transit through the secretory pathway to the plasma membrane where virus budding occurs [1]. Budding is known to require the specific interaction of the E2 cytoplasmic domain with the nucleocapsid and the formation of the envelope protein lattice (reviewed in [1,4]). In contrast, relatively little is known about the role of host proteins in alphavirus exit. Host cell membrane proteins are excluded from the site of budding and the released virus particle [1,11]. Although many enveloped RNA viruses such as retroviruses, filoviruses, and paramyxoviruses use the host cell ESCRT (Endosomal Sorting Complex Required for Transport) machinery to promote budding (reviewed in [12]), the alphavirus exit pathway is ESCRT-independent [13].

Over the past few years, a host protein known as tetherin has been shown to be an important restriction factor that inhibits the release of a number of enveloped viruses. Tetherin is encoded by an interferon-stimulated gene [14], and is officially termed BST2 and also known as CD317 or HM1.24. Tetherin inhibits the production of retroviruses such as HIV-1 and HIV-2 [15,16], herpesviruses such as Kaposi’s sarcoma-associated herpes virus [17], filoviruses such as Ebola virus [18], and rhabdoviruses such as vesicular stomatitis virus (VSV) [19]. Tetherin is a dimeric membrane protein with a unique topology consisting of a short N-terminal cytoplasmic domain, a transmembrane domain, an ectodomain that forms an extended alpha-helical rod, and a C-terminal domain containing a glycosylphosphatidylinositol (GPI) anchor [20,21,22,23,24]. Mature tetherin localizes to the plasma membrane, various endosomes, and trans-Golgi compartments [24,25,26]. Tetherin restricts virus particle release through the incorporation of one of its membrane anchors into the nascent virus particle, while the other anchor remains in the host cell membrane [27]. The completed virus particle is thus prevented from being released from the cell, and may be endocytosed and degraded [15,28,29]. In response to this restriction, viruses have evolved tetherin antagonists such as the HIV-1 Vpu protein, which acts to down-regulate the level of tetherin and thus rescue the release of progeny virus particles [29]. Recent studies demonstrate that human tetherin has two isoforms, long and short, which both inhibit the release of HIV-1 but show differential sensitivity to Vpu [30,31,32].

While tetherin can interact with many enveloped viruses, the structure of the alphavirus particle and its exclusion of host membrane proteins suggested that tetherin might not restrict alphavirus release. Here, we show that exogenous expression of human tetherin in an HEK293 cell system significantly inhibited the release of the alphaviruses SFV and CHIKV. Further studies showed that, in contrast to exit, primary SFV infection was unaffected. While both isoforms of tetherin inhibited VSV release, SFV release was only inhibited by the long isoform.

## 2. Materials and Methods

### 2.1. Viruses and Plasmids

The CHIKV vaccine strain 181/25 was provided by Dr. Robert Tesh (University of Texas Medical Branch at Galveston, TX, USA). The SFV strain used in these experiments was a well-characterized plaque-purified isolate [33]. Wild type VSV (Indiana strain) is a well-characterized plaque-purified isolate [13]. All virus stocks were propagated on BHK-21 cells and the titer determined by plaque assays on BHK-21 cells.

FLAG-tagged Ebola VP40 plasmid (pCAGGS-FLAG-VP40) was provided by Dr. Paul Bates (Perelman School of Medicine, University of Pennsylvania, PA, USA) [18,30]. The codon-optimized DENV structural polyprotein-encoding plasmid for production of virus-like particles (VLP) was a gift from Dr. Beatrice Nal (Hong Kong University-Pasteur Research Centre, Hong Kong) [34]. To generate the tetracycline-inducible RuV structural protein expression plasmid, *i.e.*, pcDNA.4/TO RuV-24S, the region encoding the RuV structural polyprotein was PCR-amplified from the M33 infectious RuV clone, a gift of Dr. Tom Hobman (University of Alberta, Edmonton, Canada). The following primers were used: forward 5' -AGAGAAAGGTTAATCTCCACGACGCTGAC- 3' and reverse 5' -AGAGAGAATTCTATGCAGCAACAGGTGC- 3'. The amplified subgenomic fragment was then inserted in pCDNA4/TO using the *Hin*dIII and *Eco*RI restriction sites.

Plasmids encoding the human tetherin constructs AU1-tagged tetherin, wild-type (WT), or the long or short tetherin isoforms were gifts from Dr. Paul Bates [18,30]. The AU1-tagged tetherin cDNA was amplified from the parental plasmid and inserted into pcDNA4/TO (Invitrogen, Carlsbad, CA, USA) using the *Kpn*I and *Xho*I sites. The WT or long- or short tetherin plasmids were digested with *Kpn*I and *Xho*I and subcloned into the pcDNA4/TO vector. All constructs were confirmed by sequencing.

### 2.2. Antibodies

Anti-tetherin rabbit polyclonal antiserum (pAb) was a gift from Dr. Klaus Strebel (National Institute of Allergy and Infectious Diseases, NIH, MD, USA) [35]. Anti-CHIKV E1 (3D8) monoclonal antibody (mAb) was provided by Dr. Michael Diamond (University of Washington School of Medicine, St. Louis, MO, USA). SFV envelope glycoproteins were detected using anti-SFV E1/E2 pAb [36]. VSV G protein was detected using the I1 mAb as previously described [37]. DENV envelope protein was detected using an anti-DENV-E mAb [38]. Anti-RuV pAb was purchased from Meridian Life Science (Memphis, TN, USA), and anti-Beta-actin and anti-FLAG mAbs were purchased from Sigma-Aldrich (St. Louis, MO, USA).

### 2.3. Cell Lines

BHK-21 (baby hamster kidney) cells were cultured at 37 °C in Dulbecco’s modified Eagle’s medium (DMEM) containing 5% FBS, 10% tryptose phosphate broth, 100 U penicillin/ml, and 100 µg streptomycin/mL [39]. HeLa (human cervical carcinoma) cells were obtained from the ATCC and were a gift from Dr. Jonathan Backer (Albert Einstein College of Medicine, NY, USA). HeLa cells were maintained in DMEM with 10% FBS, 100 U penicillin/mL, and 100 μg streptomycin/mL at 37 °C.

T-REx HEK293 (human embryonic kidney) cells (Invitrogen) that constitutively express the tetracycline repressor were cultured in DMEM with 5 μg blasticidin/ml, 10% tetracycline-free FBS, 100 U penicillin/mL, and 100 μg streptomycin/ml at 37 °C [13]. Tetracycline-inducible (Tet-On) stable cell lines expressing specific tetherin constructs were generated by transfecting the individual expression plasmids into T-REx HEK293 cells using Lipofectamine 2000 (Invitrogen) according to the manufacturer’s instructions. Stable cell lines were then selected using 125 µg/mL Zeocin (Invitrogen) as previously described [13]. We named these inducible cell lines Tet-On AU1-tetherin cells, Tet-On WT-tetherin cells, Tet-On L-tetherin cells, and Tet-On S-tetherin cells. The Tet-On AU1-tetherin cell line is a clonal cell line, while the others are mixed cell populations.

### 2.4. siRNA Mediated Depletion of Tetherin

The Dharmacon SMARTpool siRNA targeting human tetherin or a non-targeting siRNA was reverse-transfected at a final concentration of 125 nM into HeLa cells (below passage 10; 15,000 cells/well on 96-well plate; 3.6 × 10^5^ cells/well on 6-well plate) using Lipofectamine RNAiMAX (Invitrogen) (0.25% for 96-well plate; 0.5% for 6-well plate). Cells were incubated for a total of 72 h prior to subsequent experiments.

### 2.5. Analysis of Tetherin Expression

For Western blot (WB) analysis, cells were lysed in buffer containing 1% Triton X-100 and protease inhibitors, reduced and analyzed by SDS-PAGE and WB using the pAb against tetherin [30,35]. To detect specific isoforms of tetherin in cell lysates, reduced samples were treated with PNGase F (New England Biolabs, Ipswich, MA, USA) according to the manufacturer's protocol, and then analyzed by SDS-PAGE and WB.

### 2.6. Virus and VLP Release Assays

The indicated Tet-On tetherin cells were treated with 1 µg tetracycline/mL or control medium for the indicated time. The cells were then infected for 1 h at 37 °C with the indicated virus at a MOI of 10 pfu/cell in medium A (minimal essential medium containing 100 U penicillin/mL, 100 µg streptomycin/mL, 0.2% bovine serum albumin, and 10 mM HEPES, pH 7.0). The cells were washed three times and an aliquot of the medium collected to determine the input virus still present after the washes. Cells were then cultured in medium A for 6 h (SFV and VSV) or 9.5 h (CHIKV) at 37 °C, conditions that primarily assess single cycle virus infection. The culture medium and cell lysates were then harvested and the nuclei were pelleted. Medium samples were cleared by centrifugation at low speed (~500 × *g*, 5 min). The infectious virus in an aliquot of the culture medium was quantitated by plaque assays on BHK-21 cells [40], subtracting any input virus titer. In parallel, virus particles in aliquots of the same samples were quantitated as follows: the secreted virus particles were pelleted through a 20% sucrose cushion by centrifugation at 35,000 rpm in an SW41 rotor for 3 h at 4 °C. Pelleted viral particles were resuspended in TN buffer (100 mM NaCl, 50 mM Tris-HCl, pH 7.4). Lysate and medium samples were analyzed by SDS-PAGE and WB.

For virus-like particle (VLP) release assays, constructs engineered to express the Ebola virus VP40 or DENV prM-E proteins were transfected into Tet-On AU1-tetherin cells using Lipofectamine 2000 and following the manufacturer’s protocol. Tetracycline was added 5 h post-transfection to induce tetherin expression. For the RuV VLP release assay, a different approach was utilized since the plasmid expressing RuV Capsid-E2-E1 (pcDNA.4/TO RuV-24S) was regulated by tetracycline. HEK293T cells were transfected with an AU1-tetherin-expressing construct (pCB6-AU1-BST2M2, not inducible by tetracycline) or a control plasmid along with the plasmids expressing the RuV structural proteins or Ebola VP40. At 5 h post-transfection, tetracycline was added to induce expression of the RuV proteins. Cell lysates and VLP secreted into the culture media were harvested at 48 h post-transfection. Supernatants were first clarified by low speed centrifugation (~500 × *g*, 5 min) and subsequently pelleted through a 20% sucrose cushion by centrifugation at 4 °C in an SW41 rotor at 18,000 rpm for 1 h (Ebola VLP) [18] or 35,000 rpm for 3 h (rubella and dengue VLP) [41]. Samples were analyzed by SDS-PAGE and WB, and were reduced prior to loading for the RuV samples.

### 2.7. Quantitation of Release Efficiency

WB data generated from the release assays were quantitated using an Odyssey Infrared Imaging system and Odyssey InCell Western software (LI-COR Biosciences, Lincoln, NE, USA). The efficiency of particle release was calculated by determining the ratio of a viral protein in the supernatant to that protein in the cell lysates, and expressed relative to the control lacking tetherin expression.

### 2.8. Immunofluorescence Analysis

For immunofluorescence analysis, cells were fixed using 3% paraformaldehyde and stained using the indicated antibodies. Nuclei were stained using Hoechst. Fluorescence images were captured using a Zeiss Axiovert 200M microscope.

### 2.9. Statistics

Student’s *t*-test was calculated using Microsoft EXCEL to define statistical significance of data (≥3 independent experiments). The symbol * is used to indicate a statistical significance of *p* < 0.05.

## 3. Results

### 3.1. Development and Validation of an Inducible Tetherin-Expressing Cell Line

**Figure 1:**
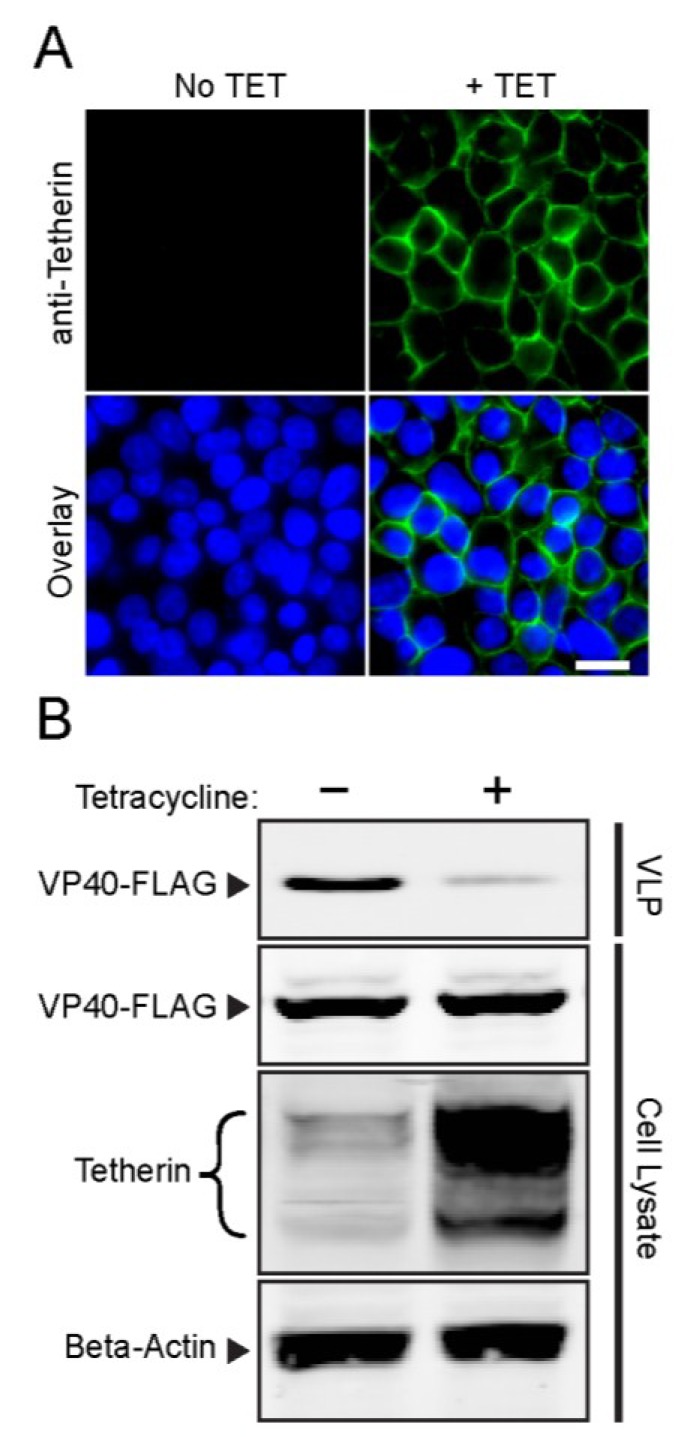
**Validation of Tet-On AU1-tetherin cells.** (**A**) Induction of tetherin expression. Tet-On AU1-tetherin cells were incubated in the presence or absence of tetracycline (TET) for 16 h. Cell surface tetherin was detected by immunostaining of fixed, non-permeabilized cells with a tetherin-specific pAb (green) and nuclei were counterstained using Hoechst (blue). Epifluorescence microscopy images are representative of three independent experiments. Bar = 20 µm; (**B**) Ebola VLP release assay. Tet-On AU1-tetherin cells were incubated with or without tetracycline for 16 h and then transfected with an Ebola VP40-FLAG-expressing plasmid. At 48 h post-transfection, VLP were pelleted and cells were lysed, and proteins detected by SDS-PAGE and WB using anti-FLAG mAb, anti-tetherin pAb, and anti-Beta-actin mAb (loading control).

While IFN treatment can trigger expression of tetherin in human cell lines, expression of many other IFN-regulated antiviral proteins can be induced or enhanced at the same time. In addition, the alphavirus infection cycle in mammalian host cells is very rapid, with robust release of progeny virions detected as soon as ~4–6 h postinfection [42]. Therefore, a timed and uniform expression system that is IFN-independent would be helpful to define the specific role of tetherin in alphavirus release. HEK293 cells are known to express very low levels of endogenous tetherin (as listed in Human Protein Atlas [43]). Using a previously reported approach [13], we developed a clonal HEK293 cell line that inducibly expresses AU1-tagged human tetherin (as described in reference [18]). The resultant Tet-On AU1-tetherin cells were treated with tetracycline-containing or control medium for 16 h and fixed with 3% paraformaldehyde. Immunofluorescence analysis demonstrated abundant tetherin protein on the plasma membrane following tetracycline (TET) induction, and, as expected, no detectable tetherin in the absence of TET treatment (Figure 1A).

To functionally examine the Tet-On AU1-tetherin cells in virus exit, we first tested them using an Ebola virus VLP system. The Ebola matrix protein VP40 generates VLP whose release is efficiently inhibited by tetherin [18,44]. Tet-On AU1-tetherin cells were induced by TET treatment for 16 h and then transfected with a FLAG-tagged Ebola VP40 (VP40-FLAG) construct. VLP were collected 48 h post-transfection. SDS-PAGE and WB analysis showed abundant expression of tetherin in the TET-treated cells, and strong inhibition of VP-40 VLP release (~90% reduction compared to untreated cells) (Figure 1B).

**Figure 2:**
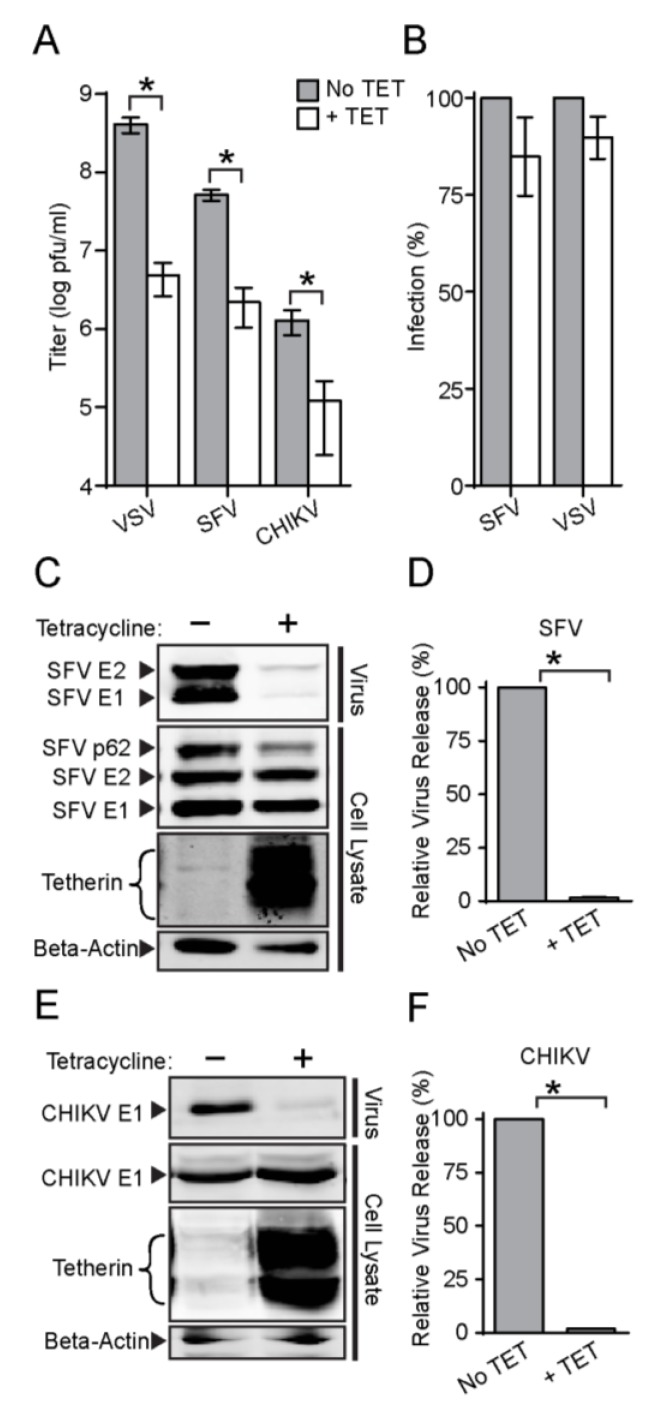
**Tetherin expression inhibits the release of infectious SFV and CHIKV.** (**A**) Tet-On AU1-tetherin cells were incubated with or without tetracycline for 16 h and then infected with VSV, SFV, or CHIKV at a MOI of 10 for 7 h (VSV and SFV) or 10.5 h (CHIKV) to assess single cycle virus infection. Infectious progeny virus was titered and graphed in a log plot. The symbol * is used throughout to indicate a statistical significance of *p*<0.05; (**B**) Primary virus infection assays. Tet-On AU1-tetherin cells were incubated with or without tetracycline for 16 h and infected with SFV (MOI = 2) or VSV (MOI = 0.04), multiplicities that allowed quantitation of primary infection. Cells were cultured overnight in medium containing 20 mM NH_4_Cl, and primary virus infection was quantitated and normalized to that of untreated cells. Graphs represent the mean and SD of three independent experiments. (**Panels C**–**F**). Virus particle release was determined by infecting cells with SFV (C, D) or CHIKV (E, F) as described in panel A. The secreted virus was pelleted and the cells were lysed, and the indicated viral and cell proteins were quantitated by SDS-PAGE and WB as in Figure 1B. The efficiency of virus particle release is shown in panels D and F, where the graphs represent the mean and standard deviation (SD) of three independent experiments. Note in the WB the SFV pAb detects p62, E2 and E1, while the CHIKV mAb detects E1.

Tet-On AU1-tetherin cells were also tested for release of infectious VSV, which has been reported to be inhibited by tetherin [19]. Cells were induced by TET treatment for 16 h and infected with VSV. At 7 h post-infection, the supernatants were harvested and released progeny virus was titered by plaque assay. In agreement with the previous report [19], we observed that production of infectious VSV particles was significantly decreased (~85-fold reduction) by tetherin expression (Figure 2A). Together these results demonstrate the validity of our inducible tetherin system for testing inhibition of VLP or virus release.

### 3.2. Tetherin Inhibits Release of SFV and CHIKV

We next tested the effect of tetherin on production of two different alphaviruses, SFV and CHIKV. Cells were induced by TET treatment for 16 h prior to virus infection. The progeny virus titers of both SFV and CHIKV were significantly decreased by tetherin expression, with reductions of ~24 fold and ~11 fold, respectively (Figure 2A).

**Figure 3:**
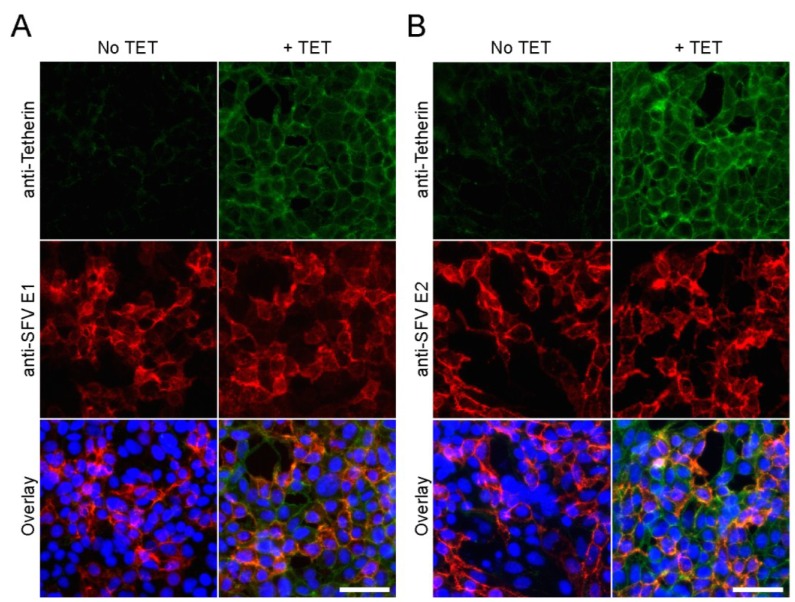
**Effect of tetherin on SFV envelope protein traffic.** Tet-On AU1-tetherin cells were incubated with or without tetracycline for 16 h and infected with SFV (MOI = 2). Samples were fixed at 16 h post-infection. Cell surface expression of SFV E1 (**A**) or E2 (**B**) was detected by immunofluorescence using mAbs to E1 or E2 (Red). Tetherin (Green) and nuclei (Blue) were detected as in Figure 1A. Representative example of two experiments. Bars = 50 µm.

Recent reports indicate that tetherin might also influence virus entry. For instance, human cytomegalovirus can exploit tetherin to enhance its entry into host cells [45]. The cell surface pool of tetherin is internalized via clathrin-mediated endocytosis, the pathway used for productive alphavirus entry [8,25]. We therefore tested if tetherin expression could affect alphavirus or rhabdovirus entry and primary infection. Tet-On AU1-tetherin cells were induced with TET for 16 h, infected with SFV or VSV, and cultured overnight in the presence of 20 mM NH_4_Cl to block secondary infection [46]. Infected cells were scored by immunostaining, and the percentage infected cells was compared to that of non-induced cells. Tetherin expression did not cause a significant difference in primary infection by either SFV or VSV (Figure 2B). Immunofluorescence experiments confirmed that the alphavirus E1 and E2 envelope proteins were both efficiently transported to the cell surface in the tetherin-expressing cells (Figure 3). To determine if tetherin inhibited the exit of alphavirus particles, we induced tetherin expression for 16 h, infected the cells with SFV or CHIKV, and analyzed virus particles released in the medium and virus protein expression in the cells by Western blot (WB). The release of both SFV (Figure 2C,D) and CHIKV (Figure 2E,F) was inhibited by tetherin expression. As predicted from Figure 2B, synthesis of the SFV or CHIKV envelope proteins was not significantly affected by tetherin (Figure 2C,E). When normalized to protein expression levels, the relative release of SFV (Figure 2D) and CHIKV (Figure 2F) was decreased by ~98% by tetherin expression in our inducible cell line. Together, these results indicate that tetherin specifically affects alphavirus exit.

### 3.3. Effect of Tetherin on the Release of Rubella and Dengue Virus

The rubivirus rubella virus (RuV) and flaviviruses such as dengue virus (DENV) share some structural similarities with alphaviruses [47,48]. In particular, the viral membrane is entrapped between an inner capsid layer containing the plus-sense RNA genome, and an outer shell composed of viral envelope proteins including a class II fusion protein. The viruses differ in their budding sites, with RuV budding into the Golgi compartment [49] and DENV budding into the ER [50]. VLP can be efficiently produced by expression of the RuV structural (capsid + envelope) proteins or the DENV envelope proteins [34,51]. Given the slow growth cycle of these two viruses, we used VLP to test the effect of tetherin on particle release.

**Figure 4:**
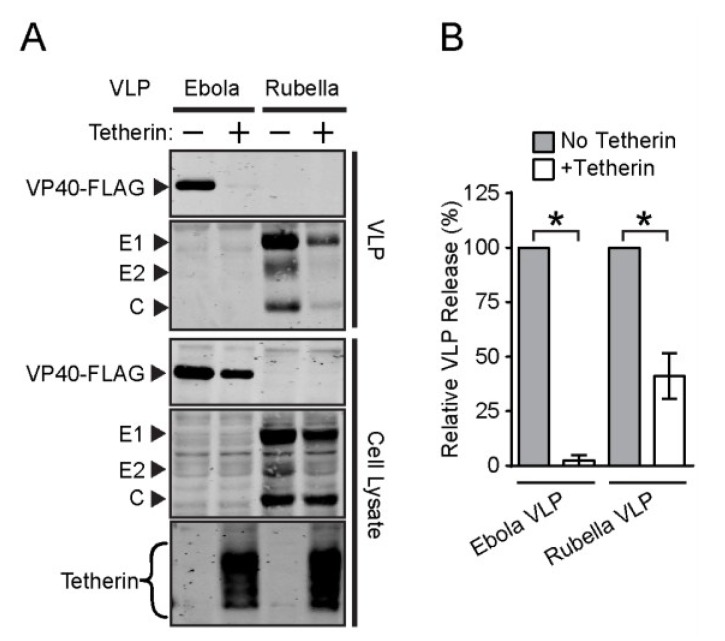

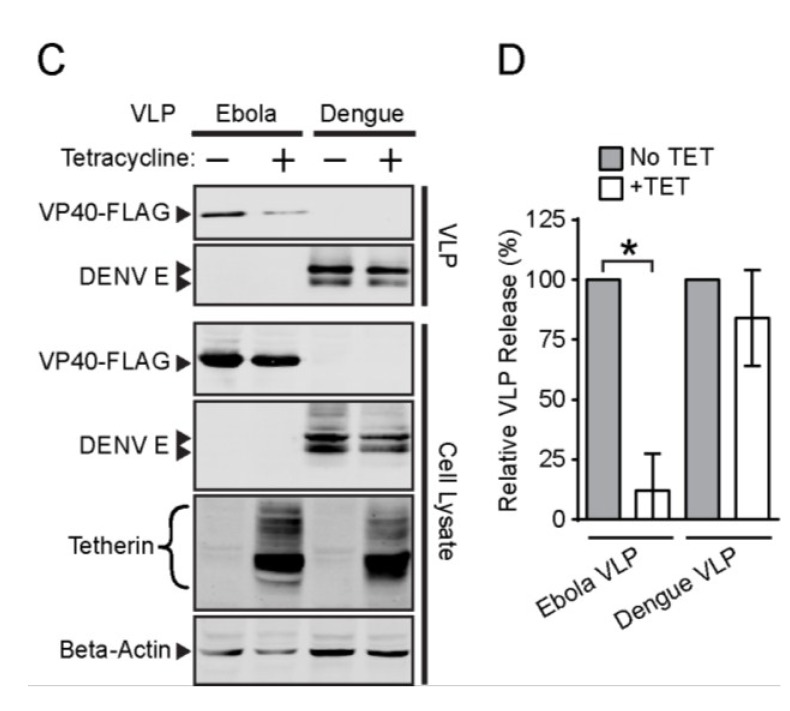
**Tetherin moderately attenuates Rubella VLP release but not Dengue VLP release.** (**A** and **B**) Rubella VLP release assay. HEK293T cells were cotransfected as indicated with plasmids expressing the Rubella virus structural proteins or Ebola VP40-FLAG and a tetherin-expressing construct (+) or empty vector (−). At 48 h post-transfection, VLP were pelleted, cells were lysed, and proteins in the samples quantitated by SDS-PAGE and WB; (**C** and **D**) Dengue VLP release assay. Tet-On AU1-tetherin cells were transfected with plasmids expressing the dengue virus envelope proteins or Ebola VP40-FLAG, and tetracycline added as indicated at 5 h post-transfection. At 48 h post-transfection, VLP were pelleted, cells were lysed, and proteins in the samples quantitated by SDS-PAGE and WB. Panels B and D show the efficiency of VLP release. The graphs represent the mean and SD of three independent experiments.

We compared the effect of exogenous tetherin on the release of RuV VLP *vs.* Ebola VLP. As the expression of our RuV structural protein construct is regulated by TET, the Tet-On AU1-tetherin cells were not suitable for this experiment. We therefore cotransfected an AU1-tagged tetherin-expressing construct plus either the RuV structural protein plasmid or the Ebola VP40-FLAG plasmid into HEK293T cells. TET was added 5 h post-transfection to trigger RuV structural protein expression. The VLP and cell lysates were harvested at 48 h post-transfection and analyzed by SDS-PAGE and WB (Figure 4A,B). In agreement with the results in Figure 1 and Figure 2, tetherin co-expression produced a ~98% decrease in Ebola VLP release. In contrast, RuV VLP release was decreased by ~59%, suggesting a moderate sensitivity to tetherin-mediated restriction.

To test DENV inhibition, we transfected Tet-On AU1-tetherin cells with plasmids encoding either the DENV prM-E envelope proteins or Ebola VP40-FLAG. Five hours post-transfection the transfection medium was replaced by fresh medium with or without TET, and at 48 h post-transfection, cells and VLP were harvested and analyzed by SDS-PAGE and WB (Figure 4C,D). As expected, tetherin expression inhibited Ebola VLP release, by ~88%. In contrast, DENV VLP release was inhibited by only ~15%, a decrease that was not statistically significant. An earlier report observed a somewhat higher level of inhibition of DENV virion production, but this result appeared to be complicated by effects of tetherin on virus primary infection [52]. Overall, our data suggest that tetherin-mediated restriction varies among members of the Togaviridae and Flaviviridae, perhaps due to their different sites of budding.

### 3.4. Endogenous Tetherin Inhibits SFV Release

**Figure 5:**
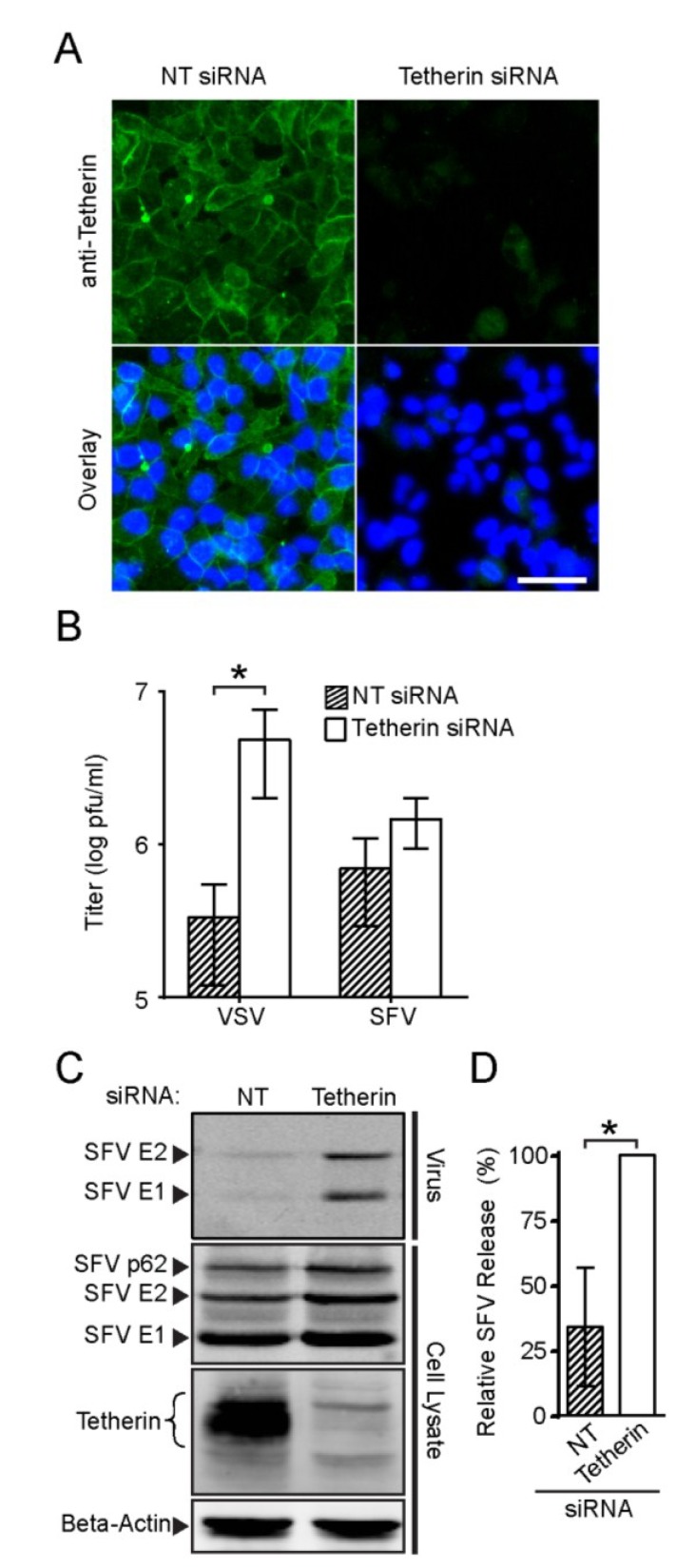
**Effect of endogenous tetherin in HeLa cells on SFV and VSV release.** (**A**) HeLa cells were transfected with non-targeting siRNA (NT) or siRNAs against tetherin, cultured for 72 h and fixed and stained as in Figure 1A. Bar = 50 µm; (**B**) HeLa cells were transfected as in Figure 5A, cultured for 65 h, and infected with VSV or SFV at an MOI of 10 for 6 h. Infectious virus in the culture medium was titered and graphed in a log plot; (**C** and **D**) HeLa cells were transfected as in Figure 5B and infected with SFV for 6 h. Released virus particles and cell lysate samples were analyzed by SDS-PAGE and WB (**C**); and the efficiency of SFV release was determined (**D**). The graphs in panels B and D represent the mean and SD of three independent experiments.

A number of cell lines including HEK293 require IFN treatment to induce abundant expression of endogenous tetherin. However, cell lines such as HeLa, Caco-2 and A-431 constitutively express tetherin at relatively high levels [43]. We tested SFV release in HeLa cells, which express endogenous tetherin at levels sufficient to inhibit HIV-1 and Ebola VLP release [18]. To compare tetherin-positive *vs.* tetherin-depleted cells, HeLa cells were transfected with a non-targeting (NT) control siRNA or with siRNAs against tetherin. Immunofluorescence analysis at 72 h post-transfection showed that cell surface tetherin was efficiently depleted by the siRNAs against tetherin as compared to the NT siRNA (Figure 5A).

We then compared the production of infectious SFV or VSV in HeLa cells transfected with the NT siRNA or the siRNAs against tetherin. Progeny virus was collected at 6 h after infection and titered on BHK cells. In agreement with a previous report [19], we observed a significant enhancement in the amount of infectious VSV in the medium (~17-fold) when tetherin was depleted (Figure 5B). Tetherin depletion also increased the production of infectious SFV (~three-fold), although this increase was below the level of statistical significance. We then evaluated the effect of endogenous tetherin on SFV release using quantitative WB analysis. Tetherin depletion significantly enhanced the efficiency of SFV particle release, with increases of two to six-fold in three experiments (Figure 5C,D). Thus, endogenous levels of tetherin could inhibit SFV release.

### 3.5. Effects of Different Tetherin Isoforms on the Release of SFV and VSV

**Figure 6:**
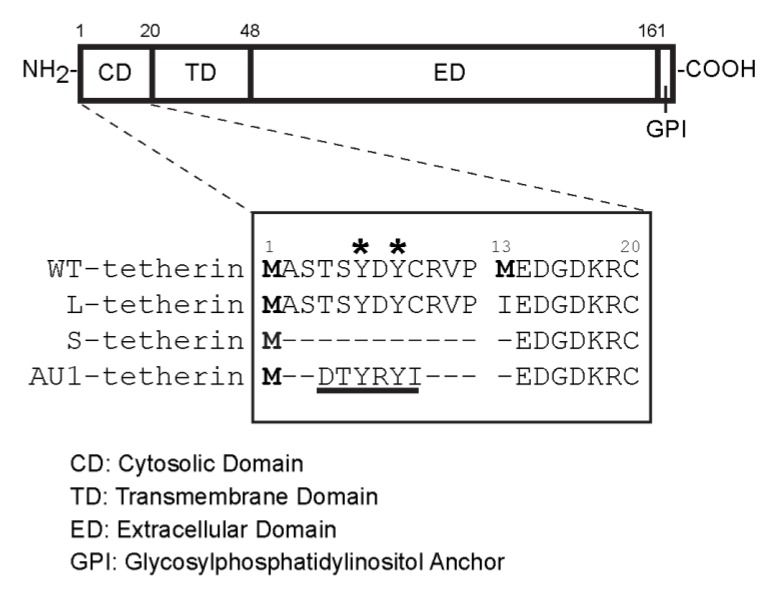
**Comparison of the cytosolic domains of different tetherin constructs.** A schematic diagram of the tetherin protein is shown at the top. The expanded sequence view below shows the forms of tetherin used in this study, indicated as WT (wild type), Long (L-tetherin), Short (S-tetherin), AU1-tagged (AU1-tagged S-tetherin), with numbers indicating residues in the WT sequence. Protein translation initiation sites are indicated by bolded methionine residues, and tyrosine residues responsible for the endocytosis of tetherin are indicated by *. The AU1 epitope tag containing a putative endocytosis motif is underlined.

**Figure 7:**
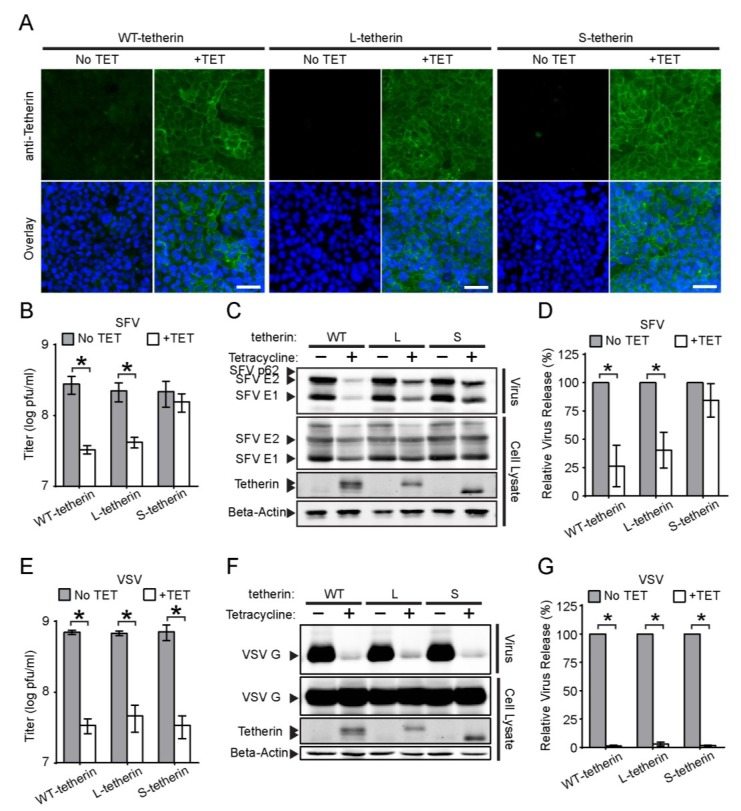
**Effects of tetherin isoforms on SFV and VSV release.** (**A**) Populations of Tet-On cells that inducibly express the long and short isoforms of tetherin (Tetherin-WT), or solely L-tetherin or S-tetherin were incubated in the presence or absence of tetracycline (TET) for 24 h. Nuclei (Blue) and cell surface expression of tetherin (Green) were detected as in Figure 1A. Bars = 50 µm; (**B**–**D**) SFV release assay. The indicated Tet-On cells were induced as in Figure 7A, and infected with SFV for 7 h. SFV particles released into the culture medium were either (**B**) titered or (**C**–**D**) pelleted for SDS-PAGE,WB and quantitation of release as in Figure 2. Tetherin isoforms were detected by WB; the gel shows the migration of the tetherin dimers. (**E**–**G**) VSV release assay, performed, titered (**E**), analyzed (**F**) and quantitated (**G**) as in **B**–**D** using a mAb to VSV-G. Graphs represent the mean and SD of three independent experiments. Note that all titers are graphed in a log plot.

A recent report described the presence of two isoforms of tetherin in all tested cell lines including HeLa cells [30]. These isoforms are due to the presence of two conserved translation initiation sites at methionine residues M1 and M13, which generate a long isoform and a short isoform (L-tetherin and S-tetherin, respectively) that differ by 12 amino acid residues within the tetherin N-terminal cytosolic domain (Figure 6). While both tetherin isoforms inhibit HIV-1 release, S-tetherin is more resistant to antagonism by Vpu and thus more efficiently inhibits WT HIV release. To determine if distinct tetherin isoforms might differentially modulate alphavirus release, we constructed Tet-On stable cells that inducibly expressed untagged versions of WT tetherin (*i.e.*, both isoforms), or only L-tetherin or S-tetherin. Immunofluorescence assays after Tet-treatment demonstrated that all of these HEK293 cell lines inducibly expressed tetherin on the cell surface (Figure 7A).

We then examined the effect of the different tetherin isoforms on SFV and VSV release. Production of infectious SFV was significantly inhibited in both the WT tetherin and L-tetherin-expressing cells (~8-fold and 5-fold, respectively) (Figure 7B). Quantitative WB assays also demonstrated significant reductions in SFV particle release, ~74% for WT tetherin and ~60% for L-tetherin (Figure 7C,D). In contrast, no significant inhibition was observed in cells expressing S-tetherin (Figure 7B–D). However, all of the tetherin isoforms reduced VSV progeny virus titers (~15–21 fold) (Figure 7E) and inhibited VSV particle release (~97%–99%) (Figure 7F,G). Thus, while all of the tetherin isoforms produced in these cells were functional, S-tetherin was specifically unable to restrict SFV release.

## 4. Discussion

A report by Atasheva *et al.* showed that VEEV infection induces the expression of a number of interferon-stimulated genes including tetherin, and that the overall growth of VEEV is inhibited by expression of murine tetherin [53]. Human tetherin causes an accumulation of CHIKV VLP on the plasma membrane as observed by scanning electron microscopy [54], and decreases production of infectious CHIKV [55]. Here, we demonstrated that tetherin specifically blocked the release of the alphaviruses SFV and CHIKV. Tetherin inhibited the production of infectious alphavirus particles during a 6 h virus infection, without significantly inhibiting virus entry into host cells, primary infection, or the production of the viral envelope proteins. Together, our data and that of others conclusively demonstrate that tetherin acts as a restriction factor to modulate alphavirus release.

The report by Cocka and Bates [30] revealed the existence of two distinct isoforms of tetherin, and together with parallel studies [31,32] demonstrated a key role of the tetherin N-terminal domain in signaling. These results prompted our investigation of the activity of the two isoforms in modulating alphavirus exit. Our results showed that the untagged short isoform of tetherin, although effective in restricting VSV release, did not significantly inhibit SFV release. In contrast, expression of mixed isoforms from WT tetherin or expression of either L-tetherin or AU1-tagged tetherin alone produced efficient restriction of both VSV and SFV release. This suggests that the N-terminal cytoplasmic domain is important in tetherin’s activity against alphaviruses.

A number of activities are associated with the twelve residue cytoplasmic domain of L-tetherin (see reference [56] for review). This domain contains a non-canonical dual tyrosine endocytosis motif (Y6 and Y8) that interacts with the clathrin AP1 and AP2 adaptor complexes [25,26]. Cell surface tetherin is internalized via clathrin-mediated endocytosis and traffics via endosomes to the trans-Golgi network. Truncation of the first 12 residues of L-tetherin significantly impairs its endocytic uptake from the cell surface [25], suggesting that internalization of the S-tetherin isoform would also be impaired. Intriguingly, insertion of the AU1 epitope tag (DTYRYI) at the N-terminus of S-tetherin confers a YXY motif, similar to the endocytic motif found on L-tetherin (Figure 6). Thus, tetherin internalization could be important for inhibition of alphavirus release. However, Vpu-deficient HIV [30] and VSV are efficiently restricted by both tetherin isoforms, suggesting that internalization is not universally required for tetherin restriction and that in its absence virus particles still remain anchored to the plasma membrane. Alternatively, it is possible that the length or composition of the tetherin cytoplasmic domain is particularly important to facilitate tethering of alphaviruses, but not VSV, to the plasma membrane. The GPI anchor of tetherin mediates its association with detergent-resistant membrane microdomains, and tetherin’s N-terminal cytosolic domain promotes exclusion of a heterologous protein from these domains [57]. However, direct comparison of S- and L-tetherin indicates that both have similar association with membrane microdomains [57]. An important isoform difference is that only L-tetherin can trigger the nuclear factor kappa B (NF-κB)-regulated immune response [30], in keeping with the demonstrated role of the tetherin N-terminus in signaling [31,32,58]. Signaling may not play an important role in alphavirus restriction, since it is inhibited by high levels of L-tetherin expression or by co-expression of L- and S-tetherin [30], while we observed efficient alphavirus restriction under both of these conditions. However, BST2^−/−^ mice infected with CHIKV show increased systemic viral loads and decreased production of antiviral factors, suggesting that tetherin signaling can play a role at some point in alphavirus infection [55]. Further investigations will be needed to define the mechanism of differential inhibition by the two tetherin isoforms and the potential role of signaling.

The current paradigm for tetherin-mediated interception of virus release is through the incorporation of the membrane anchors of tetherin into nascent virions budding at the plasma membrane [21,27,59]. It was not clear if such a physical interaction could be affected by the structure of the virus particle. Our data support the ability of tetherin to restrict the release of highly organized alphavirus particles, even though host membrane proteins are excluded from the nascent virus particle [11]. Tetherin could block a late stage of virus assembly, or inhibit the release of completed alphavirus particles. The length of the tetherin ectodomain (~170 Å) [23,60] appears sufficient to bridge the distance between the lipid bilayer of the completed alphavirus particle [61] and the plasma membrane. While alphaviruses, rubella virus and flaviviruses all have organized envelopes with closely packed arrangements of the viral membrane proteins [6,50,62], we found that these viruses had different degrees of sensitivity to tetherin restriction. Such decreased sensitivity could be due to the location of budding, as RuV and DENV bud into the lumen of the Golgi apparatus and the ER, respectively, rather than at the plasma membrane. The final envelopment of herpesviruses occurs at the membranes of the Golgi apparatus and/or endosomal compartments [63], and similar to RuV, both alphaherpesviruses and gammaherpeviruses are sensitive to tetherin inhibition [17,64,65]. The ER is the site of membrane protein oligomerization [66], and tetherin must dimerize to be active [59,67]. Thus, the resistance of DENV VLPs to tetherin inhibition could be due to insufficient tetherin dimers during VLP budding into the ER. Tetherin antagonism by the DENV and RuV structural proteins might also influence the levels of VLP release.

Our experiments demonstrated that both endogenously- and exogenously-expressed tetherin was functional and caused efficient inhibition of VSV release. In contrast, alphavirus release was significantly less inhibited by the endogenously expressed tetherin in HeLa cells compared to the inhibition by exogenous over-expression of tetherin in 293 cells. It is possible that restriction of alphavirus release requires high levels of tetherin, and thus that the difference between the endogenous *versus* exogenous systems simply reflects their different levels of tetherin expression. HIV studies have shown that tetherin restriction is saturable under conditions of high virus particle production [59]. Alternatively, alphaviruses might antagonize tetherin, and indeed expression of the alphavirus nsP1 protein was found to promote CHIKV VLP release [54]. The level of tetherin in HeLa cells could be insufficient to overwhelm such a counteractive mechanism. It is also possible that a putative alphavirus antagonist is more effective against S-tetherin *vs.* L-tetherin, perhaps accounting for the more efficient inhibition of alphavirus release by L-tetherin.

Our work raises new questions for further investigation. It will be important to define the reasons for the differences in alphavirus inhibition by the two tetherin isoforms, and the role of viral antagonism of tetherin restriction. The broad host range of alphaviruses suggests that the species specificity of both inhibition and antagonism could be interesting areas to explore. While it seems likely that inhibition occurs by insertion of the tetherin membrane anchor(s) into the alphavirus particle, the membrane anchor(s) involved and the fate of tethered alphavirus particles are unknown. Ultimately, understanding the tetherin-alphavirus interaction may also provide insights into the overall mechanism of alphavirus exit.
